# EchoSee: An Assistive Mobile Application for Real-Time 3D Environment Reconstruction and Sonification Supporting Enhanced Navigation for People with Vision Impairments †

**DOI:** 10.3390/bioengineering11080831

**Published:** 2024-08-14

**Authors:** Broderick S. Schwartz, Seth King, Tyler Bell

**Affiliations:** 1 Department of Electrical and Computer Engineering, College of Engineering, University of Iowa, Iowa City, IA 52242, USA; broderick-schwartz@uiowa.edu; 2 Department of Teaching and Learning, College of Education, University of Iowa, Iowa City, IA 52242, USA; seth-king@uiowa.edu

**Keywords:** assistive technology, visual impairment navigation, spatial audio feedback, 3D reconstruction, 3D localization and mapping, human–computer interaction, rehabilitation, sensory substitution

## Abstract

Improving the quality of life for people with vision impairments has been an important goal in the research and design of assistive devices for several decades. This paper seeks to further that goal by introducing a novel assistive technology platform that leverages real-time 3D spatial audio to promote safe and efficient navigation for people who are blind or visually impaired (PVI). The presented platform, EchoSee, uses modern 3D scanning technology on a mobile device to construct a live, digital 3D map of a user’s environment as they move about their surroundings. Spatialized, virtual audio sources (i.e., virtual speakers) are dynamically placed within the digital 3D scan of the world, providing the navigator with a real-time 3D stereo audio “soundscape.” The digital 3D map, and its resultant soundscape, are continuously updated as the user moves about their environment. The generated soundscape is played back through headphones connected to the navigator’s device. This paper details (1) the underlying technical components and how they were integrated to produce the mobile application that generates a dynamic soundscape on a consumer mobile device, (2) a methodology for analyzing navigation performance with the application, (3) the design and execution of a user study investigating the effectiveness of the presented system, and (4) a discussion of the results of that study along with a proposed future study and possible improvements. Altogether, this paper presents a novel software platform aimed at assisting individuals with vision impairments to navigate and understand spaces safely, efficiently, and independently and the results of a feasibility study analyzing the viability of the approach.

## 1. Introduction

A substantial and growing number of people around the world have varying degrees of vision impairment, ranging from easily correctable impairments to complete blindness [1]. The anticipated growth comes as a result of demographic shifts due in part to aging populations [2]. Referred to collectively as people who are blind or visually impaired (PVI), these individuals experience a variety of challenges, among which is the safe and efficient navigation of unknown environments. PVI have adopted a variety of assistive technologies throughout history and into the modern day to augment their capacity to navigate and complete daily tasks [3]. Such navigational aids can be broadly separated into two categories, agent- and non-agent-based approaches. Agent-based approaches incorporate aid from other people, either as in-person or remote assistants, or from trained animal assistants (i.e., guide dogs). Non-agent-based approaches include the wide range of devices and techniques that do not require the direct involvement of other beings.

Arguably, the oldest navigational aid that PVI have used is the help of another person, typically family and friends, or perhaps hired assistants. Direct, personal assistance, to be guided through unfamiliar environments by trusted individuals who can clearly communicate and sympathize, is a powerful and effective tool for PVI. However, this requires the physical presence of another person and not all PVI have access to such assistance. Several services, such as Be My Eyes [4] and Aira [5], have attempted to ameliorate this problem of access by providing remote assistance services. These technologies connect PVI to sighted volunteers and professionals, sometimes through video calls, so that they can receive help completing everyday tasks, including navigation.

Guide dogs have also been used by PVI throughout history, although they did not exist in something like their current form in America until the end of the 1920s [6]. Since their modern introduction, guide dogs have been gaining cultural and official acceptance, eventually resulting in their use within the United States being federally protected by the Americans with Disabilities Act in 1991 [7].

Relying on another being, however, may introduce limitations on practical independence. There may be PVI who do not have friends and family to help or who are unable to afford a hired assistant or guide dog. It may also be impractical to have an assistant physically present in various contexts. Although some of these drawbacks are overcome by remote assistants, such services are not without their own downsides, perhaps most importantly the necessity of internet or cellular connectivity to use them, followed by the cost and time interval that may be required for a response to be received.

Non-agent-based technologies are able to mitigate some of the deficiencies that are present in agent-based approaches. A variety of these technologies has been developed to aid PVI in accomplishing various different tasks, with many of them focusing on improving navigational independence. Canes are possibly the oldest assistive device (in their current form known as “white canes”), which serve primarily as an extension of tactile input, allowing PVI to feel objects and surfaces at a safer distance, providing warning of nearby obstacles. However, the effective range of the white cane is relatively limited and information is only delivered sparsely. These inherent restrictions have prompted a variety of efforts to either improve, augment, or replace the white cane with an electronic device.

These devices, and others with similar aims, are typically referred to as Electronic Traversal Aids (ETAs) or Sensory Substitution Devices (SSDs), which emphasizes that the chief goal of such devices is to assist PVI by augmenting their senses (principally their hearing and touch). A large number of such devices were commissioned and investigated in the aftermath of the Second World War, aiming to help “the average blind person to find their way about with more ease and efficiency than with the aid of the traditional cane or the seeing-eye dog” [8]. These initial prototypes were often composed of multiple components, typically a handheld emitter, a shoulder-slung pack with control circuitry and battery, and headphones for output. Several scanning signals and feedback modes were investigated, including both light and sound sources, with tactile and auditory outputs always indicating distances to single points within the environment. In the decades following this representative early work [8], derivative and novel devices were produced using similar principles. Efforts were also made to quantify the potential benefits these devices offered to PVI in the form of accompanying user studies [9,10,11,12].

Along with the development and characterization of these approaches to assistive devices, researchers have investigated the capacity of humans to interpret environmental reverberations of sound to extract spatial information about their surroundings. Though this capacity has been noted throughout history, formal studies have been performed only more recently [13,14,15]. Subsequent research efforts were undertaken to quantify the limits of this “biosonor” in terms of which emitted sounds result in better information acquisition, how well distance and angular resolution (also termed lateralization) can be discerned, and also the degree to which other kinds of information can be determined (e.g., material, texture, etc.) [16,17,18,19,20,21,22,23,24,25].

The continued improvement in modern computing technologies has enabled increasingly efficient processing for tasks including accurate acoustic simulations and the simulation of fully virtual environments. Many researchers have used this expanded processing power to apply virtual acoustics and virtual environments to the task of studying human echolocation and how PVI perceive virtual spaces [26,27,28,29,30,31,32,33,34,35,36]. Some researchers have moved further into virtualization techniques by investigating the transfer of real-world navigation skills into virtual environments [37,38,39,40,41,42] and vice versa [43]. Many of these technologies use virtual reality (VR) or augmented reality (AR) headsets, with other supplementary devices, as the operational platforms since these technologies are designed to provide motion tracking and spatial audio within virtual environments.

Although navigation is often the primary goal and echolocation approaches demonstrate promise, not all devices designed to assist PVI are geared directly toward echolocation or its use for navigation. Several technologies have aimed to support PVI by providing different kinds of information. One such system uses an augmented reality headset “HoloLens” from Microsoft to detect and identify people in a scene and inform the user via per-identified-person spatialized audio feedback [44]. Another technology, combining mobile imaging with virtual reality techniques, has been successful in offering magnified views to low vision users [45]. That said, such headset-reliant implementations may not improve visual motor function or mobility [45], may be relatively expensive, and may fatigue the user if worn for too long. Practically, it is also one more additional item that PVI must carry with them or wear.

Prior work has been conducted that encodes one’s spatial surroundings into sound. For example, in [46], various types of audio signals were used to alert and potentially guide the user. Another work in this area incorporated two cameras into a stereovision, head-mounted system worn by the user [47]. The two cameras worked together to reconstruct the 3D scene in front of the user. A “sonification algorithm” was then used to encode the 3D scene into an audio signal that could be played back to the user. While its users were able to employ the developed prototype for spatial orientation and obstacle avoidance [47], the head-worn device was somewhat bulky and required a 10 m cable tether to a PC (or the wearing of a special laptop backpack) and was limited to processing at approximately 10 frames per second, thus restricting its practical viability.

In 2017, researchers incorporated a depth-imaging camera (for indoor and low-light settings) and a stereovision system (for outdoor settings) directly into specially designed headgear [48]. These imaging systems were then used to perform 3D reconstruction, 3D ground detection and segmentation, and ultimately, detection of objects within the environment. While it provided an advantage by extending the scope in which such devices could be used, the user was still required to wear a tethered headset. This work [48] also notes reasons why consumer-grade assistive systems have not experienced wide adoption by PVI, such as form factor and the lack of efficient training programs.

**Figure 1:**
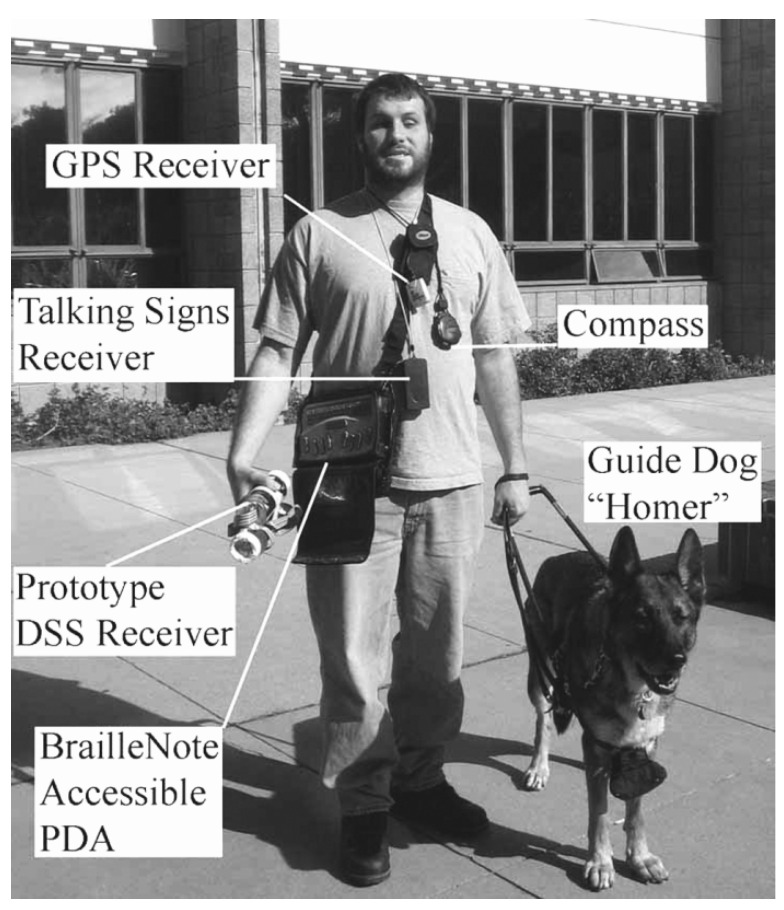
An example of a PVI making use of various assistive technologies [50]. “A blind pedestrian is using a guide dog and five technologies for navigation. This figure illustrates the need for an integrated navigational system. The guide dog aids with mobility and obstacle avoidance. The compass provides the user with heading information when stationary. The GPS [Global Positioning System] receiver integrates with a GIS [Geographic Information System] database (digital map) to provide position and heading information during outdoor navigation. The talking signs receiver gives orientation cues by identifying the direction and location of important landmarks in the environment. The digital sign system (DSS) receiver picks up barcodes from signs and sends them to a database to facilitate indoor navigation. The BrailleNote accessible computer represents the ‘brain’ of the system, allowing Braille input and speech and Braille output. In theory this device could serve as the hub to which all other technologies interface”.

Many of the aforementioned technologies have been implemented using equipment that is relatively obtrusive, many requiring headsets, backpacks, or both. If a PVI were to use a set of these devices covering the spectrum of assistance modes, they could quickly become prohibitively encumbered. A demonstrative example of a PVI equipped with a suite of assistive technologies is shown in Figure 1. Equipped in this manner, a PVI is also likely to be much more noticeable, which may bring unwanted attention or create social barriers [49].

There are some approaches that operate using multipurpose devices that many PVI already own (i.e., smartphones) such as The vOICE [51,52] and Microsoft’s SeeingAI app [53]. The vOICE is an image and video sonification technology with mobile and desktop implementations (that can also make use of glasses-mounted cameras or augmented reality headsets) whereby “images are converted into sound by scanning them from left to right while associating elevation with pitch and brightness with loudness” [52]. SeeingAI provides a suite of features for iOS devices including in situ speech-to-text, person recognition (with facial expression description), brightness sonification, “an experimental feature to describe the scene around you”, and another experimental feature that allows users to place waypoints into the scene and navigate to them with a virtual-white-cane-like haptic range detector and spatial audio. These approaches have potential, but there are limitations. The vOICE relies on purely 2D image processing, while SeeingAI requires an internet connection for several of its services. There is a great opportunity in the arena of ETAs and SSDs for improvements in the application of modern mobile devices, which already combine within them many of the capabilities of the several devices shown in Figure 1. Mobile devices are increasingly being equipped with depth sensors of various capacities such as the LiDAR (light detection and ranging) scanner on newer iPhones.

This paper presents EchoSee, a novel assistive mobile application that leverages modern 3D scanning and processing technologies to digitally construct a live 3D map of a user’s surroundings on an iPhone as they move about their space. EchoSee uses real-time (∼60 fps) onboard processing to generate a 3D spatial audio soundscape from this live map, which is played to the user via stereo headphones. Additionally, the methodology and results of a preliminary user study are presented. This study assesses the effectiveness of EchoSee by means of an adapted alternating treatment design (ATD) with one familiarization and two trial conditions consisting of (1) sighted navigation, (2) blindfolded and unassisted navigation, and (3) blindfolded and EchoSee-assisted navigation of a permuted obstacle course. Several specific research questions were pursued during this study:

- What is the frequency with which a participant encounters an obstacle when blindfolded and assisted by the application compared to when blindfolded and unassisted?
- How quickly can a participant traverse the obstacle course blindfolded with the assistance of EchoSee as compared to without it?
- How much “seeking” behavior (head turning off axis from the direction of travel) occurs in participants during obstacle course traversal with the assistance of the application as compared to when blindfolded or unassisted?

Overall, it is the driving goal of EchoSee to enhance the ability of PVI to navigate safely and efficiently, thereby improving their functional independence. A summary table containing several representatives from the wide range of prior research work, applications, services, and techniques is presented as Table 1. The table contains the title of the technology or service, or the author and publication year of the paper, a short summary of the entry, as well as various additional descriptions and whether it meets a given criteria. The last row in the table highlights how EchoSee compares to the selected approaches.

The remainder of this paper is structured as follows. Section 2 details the materials and methods used in developing EchoSee and conducting the user study. Section 3 presents the results from both developmental testing and the user study. Section 4 discusses the implications of these results, addressing EchoSee’s strengths, limitations, and potential real-world applications. Finally, Section 5 concludes the paper, summarizing key findings and future directions for this assistive technology. A preliminary version of this work was published in the proceedings of the 3D Imaging and Applications conference at the Electronic Imaging Symposium 2023 [54].

## 2. Materials and Methods

### 2.1. Hardware and Software

**Figure 2:**
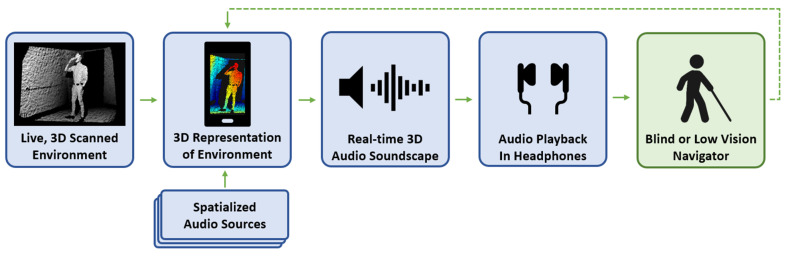
Block diagram of the proposed mobile application. First, the initial environment scan is made, creating a 3D map of the scene. Second, the 3D map is processed using the underlying game engine and spatialized audio sources are placed according to raycasts at the specified angular offsets. Third, the soundscape is synthesized from the virtually placed audio sources. Fourth, this soundscape is played to the user with stereo headphones. Fifth, the user interprets the audio information and uses it to navigate through their environment. As the user moves about their environment, the 3D map, virtual audio sources, and soundscape are all updated in realtime.

Inspired by echolocators who can navigate by making and interpreting the reverberations of short “clicking” sounds [14], this work describes a novel assistive application platform to leverage modern 3D scanning technology on a mobile device to digitally construct a 3D map of a user’s surroundings as they move about a space. Within the digital 3D scan of the world, spatialized audio signals are placed to provide the navigator with a real-time 3D stereo audio “soundscape”. As the user moves about the world, the soundscape is continuously updated and played back within the navigator’s headphones to provide contextual information about the proximity of walls, floors, people, and other features or obstacles. This approach is illustrated in Figure 2.

To allow for the on-demand creation of virtual environments and for the realistic simulation of spatialized audio, the mobile application was implemented in the Unity game engine [55]. Newer Pro models of the iPhone and iPad were targeted for this research as they are equipped with a built-in, rear-facing LiDAR scanner. With Apple’s ARKit [56] (the underlying software development framework for Apple’s augmented reality capabilities), depth data from this LiDAR scanner can be used to produce a real-time 3D mesh reconstruction of the user’s physical environment. The ARFoundation [57] plugin was used to interface Unity with this dynamic scene generated by ARKit.

Once a digital reconstruction of the world’s geometry is established, the soundscape is created by placing an array of spatialized audio sources within the virtual world. The positions of these audio sources are determined by “raycasting.” Raycasting is performed by projecting an invisible line (a ray) into the digital reconstruction of the world in some direction. Any collision of this virtual ray with a part of the digital world’s reconstructed geometry (e.g., a wall) is detected. Once a collision point is known, a virtual audio source is placed at that location. This process can be repeated for any desired number of rays (audio sources) or initial angles of offset.

**Figure 3:**
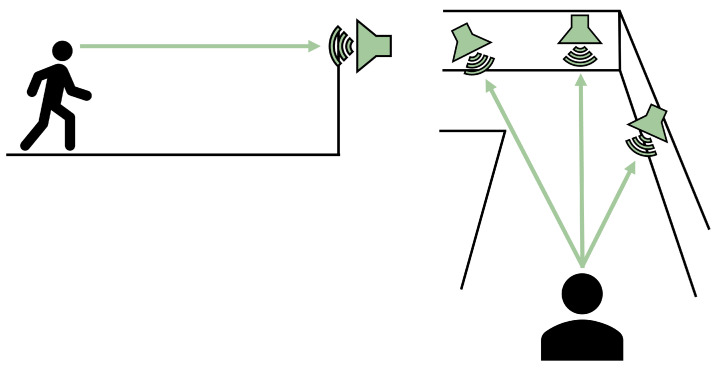
Representation of using raycasting to place audio sources. (**Left**): an audio source is placed where the ray intersects with the wall in front of the user. (**Right**): multiple rays at various angles are used to place several audio sources; this technique is used to create the spatial audio “soundscape”.

Figure 3 illustrates this approach. In this simple arrangement, navigational information is provided to the user by intensity differences in the stereo sound output from three audio sources. As shown here, the right source would have a greater audio intensity relative to the other two, indicating that it is closer to the navigator (i.e., louder in the right ear and quieter in the left ear). As the user progresses down the corridor, this right source would maintain a similar intensity, guarding the navigator against turning into the wall. In contrast, the audio from the sources ahead (the central and left sources) would continue to increase in amplitude, informing the navigator that they were approaching the end of the corridor. When the end of the corridor is reached, the navigator could rotate to determine the “quietest” direction, which would indicate the path of least obstruction. That information could then be used to decide how to proceed.

**Figure 4:**
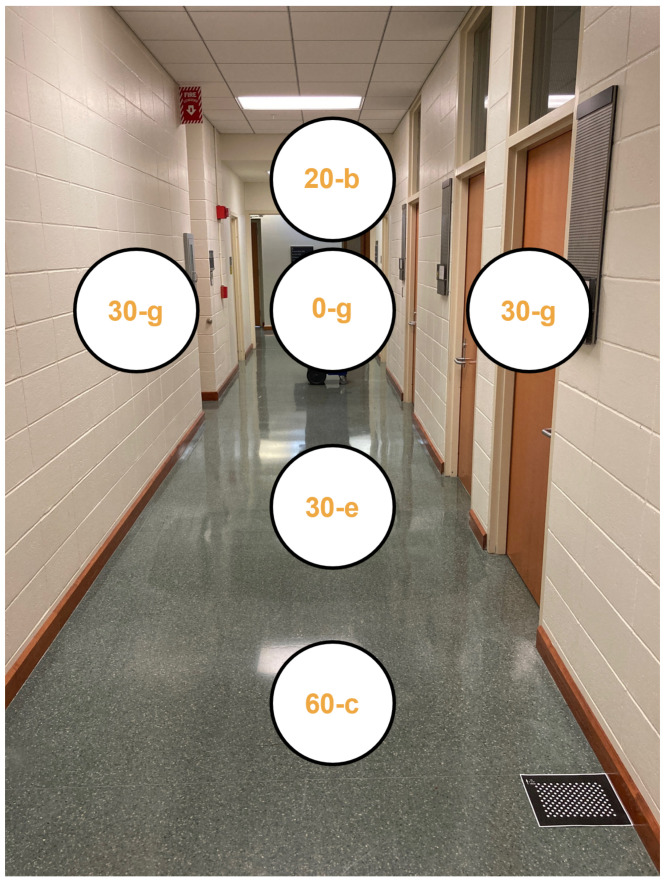
Audio source raycast configuration used by EchoSee. Each sphere is labeled with its angle of offset from the center of the field of view and the letter name of the note/pitch of the audio signal played during operation. All notes were selected from the fourth octave with each vertical level receiving its own tone (the central row of three spheres sharing the same tone). The frequencies for each level, ordered from top to bottom, are 493.88 Hz, 392 Hz, 329.63 Hz, and 261.63 Hz, respectively.

For EchoSee, raycasting was performed by a native Unity subroutine. In the presented implementation, six audio sources were used in a “t” configuration as shown in Figure 4. The left and right sources are $30^{\circ }$ offset from the center source, all three of which currently play a G4 tone. The upper source is $20^{\circ }$ offset from center and plays a B4 tone. The other two remaining sources are at $30^{\circ }$ and $60^{\circ }$ down from the center source and play E4 and C4 tones, respectively. As a user navigates, each ray is continuously re-cast into the digital reconstruction of the world and the position of the associated audio source is updated. Depending on the world’s geometry and the user’s perspective, each audio source will have a unique stereo volume intensity (related to how far it is from the user and its lateral position with respect to their head orientation). It will be these amplitude variations that produce the soundscape when spatially combined. The sounds produced by each of the audio sources that compose the soundscape can be individually adjusted (though the optimization of their combination is a limitation yet to be explored and an active area of future research). For the current implementation, audio sources have been arbitrarily configured to play the pure tones listed above. Irrespective of the specific sounds played, each audio source is spatialized by Unity within the digitized 3D environment. The resulting soundscape is then output to the user via stereo headphones. Many prior works have used different types of over-ear headphones, as they were focusing on characterizing precise audiological details. For this application, Apple AirPods Pro were adopted as they include modes for active noise cancellation and more important, ambient audio passthrough. This audio passthrough feature allows EchoSee’s soundscape to be digitally mixed with sounds from the user’s current physical surroundings, thereby not isolating them from environmental stimulus that might be crucial for safety.

EchoSee has been designed with the option of recording its position and orientation within the user’s reconstructed environment for each of the 60 frames it renders every second. The virtualized environments generated by EchoSee have an internal coordinate system that is created arbitrarily on a per-trial basis. To enable the cross-trial comparison of participants’ performance in the presented study, a registration procedure was implemented. This procedure relied on a fiducial marker—in the form of a calibration panel (one is present in the bottom right corner of Figure 4)—that was linked to a virtual object (e.g., a cube) within the EchoSee coordinate system. When the fiducial marker was observed by the system, the virtual object was automatically placed at the position corresponding to the real-world location of the fiducial marker. This fiducial marker was placed in a consistent position throughout the study trials, allowing it to define both a ground truth origin and a neutral rotation. During the execution of all trial runs, at each time step, EchoSee recorded the position and orientation of the iPhone within the virtualized 3D space created by the application. During subsequent analysis, these data were rotated and translated (registered) such that the arbitrarily initial origin of each trial was shifted and aligned to the location and orientation of the virtual object linked to the fiducial marker, thus enabling all trials and participants to be evaluated within a common coordinate system.

EchoSee was designed to operate in two modes, active and passive. In active mode, EchoSee generates the described virtualized environment and plays the audio soundscape to the user while recording its own position and orientation once per rendered frame. In passive mode, EchoSee only records its position and orientation, not providing any soundscape feedback to the user. These modes were able to be selected by the researchers at runtime.

**Figure 5:**
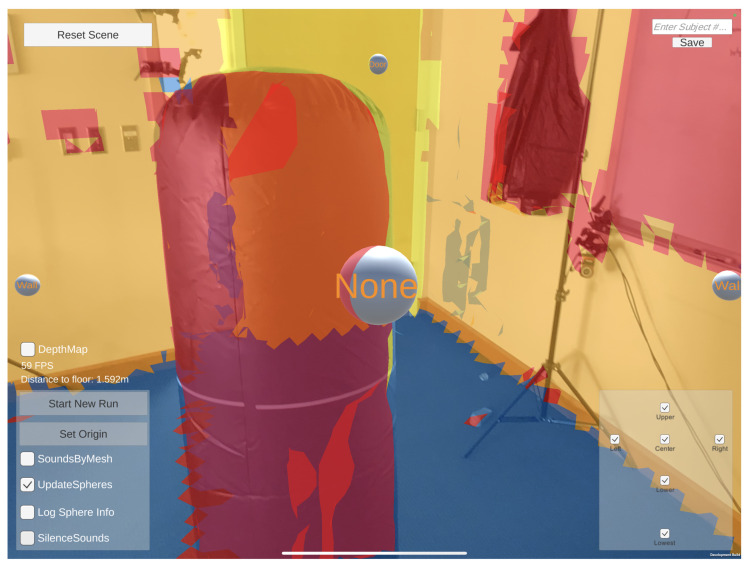
Screen capture of the EchoSee application running on an iPad Pro during late stage development. Each audio source is represented with a sphere. The spatialization of the audio sources is evident in the differing sizes of the spheres in the scene. Color overlays specify which world features EchoSee is able to automatically segment and classify (e.g., walls, floors, doors, tables).

A capture of EchoSee in its current operational state is presented as Figure 5. The capture scene contains an inflatable column (i.e., a punching bag) in the foreground and a door in the corner of the room in the background. The color texturing corresponds to the class that is assigned to the surface by ARKit [56], the visible classes are: “wall” in beige, “floor” in blue, “door” in yellow, and “other” in red. Four of the six audio sources currently used by EchoSee (center, upper, left, and right) are visible in this frame as spheres, the remaining two lower sources are outside of the field of view. The spatialization produced by the raycasting described above is apparent in the differing sizes of the spheres representing the audio sources. The most evident size difference is between the center and upper spheres. The center sphere is much closer to the capture device, shown by its much larger relative size, meaning the audio signal from the center source is playing much more loudly than those of the other sources. This louder audio signal is what would communicate to PVI the presence of a nearby obstacle in the middle of the scene. In contrast, the other sources (especially the upper one) would have quieter audio outputs indicating the increased distance to potential hazards. Together with the constructed mesh of the environment (represented by the colors overlaying the scene) and the spatialized audio sources (represented by the spheres), several interface panels are also visible in Figure 5. These panels are primarily for developmental and study use and include controls for turning on and off each of the sources (the checkboxes in the bottom right of the frame), changing system parameters such as whether to record session data, whether to play the soundscape, whether to display the raw depth map from the LiDAR sensor, etc. (visible in the bottom left). Furthermore, notable is the “Distance to floor: 1.592 m” visible at the center left of the frame, which corresponds to the top of the inflatable column whose height is 1.6 m. The elements at the upper corners of the application allow the operator to refresh the mapping of the environment (“Reset Scene” in upper left) and change the file identifier for the logging of tracking information (text box in upper right).

### 2.2. Participants

For this study, 7 participants were recruited from the local area of Iowa City, Iowa. All participants were healthy adult volunteers who reported normal hearing and normal or corrected-to-normal vision. The mean participant age was 23.2 ± 2.05 years, the male/female distribution was 5/1 with one participant declining to share age and gender. Sighted participants were chosen for this study rather than PVI for a few reasons: (1) the population was more accessible, (2) there were less risks associated with including only fully-sighted individuals, and (3) the inclusion of PVI was anticipated to have a high chance of introducing confounding effects into the study due to their presumably significant familiarity with navigation assistance technologies and the potential for predicate biases for or against a particular approach. This decision was made only for the presented feasibility study. It is anticipated that after technical and methodological refinements of the application, a broader investigation into the performance of EchoSee will be conducted where PVI would ideally compose the principle cohort of the study.

Participants completed the three conditions (sighted introduction and two blindfolded treatment conditions) in either two or three sessions lasting between 30 and 90 min each. Before each session, participants were verbally instructed or re-instructed regarding the nature of the trials to be completed, the type of obstacle course that would be navigated, the equipment that was being used, and the experimental protocol in operation. Qualitative feedback was collected from participants in the form of a perception survey at the end of their involvement in the study, any unprompted comments made regarding the EchoSee application during testing, and general questions at the end of each session.

Participant one did not complete the entire study and their partial results are not included. Only partial results were recorded during the sighted phase for participant two, so those sighted data were not reported. However, the full set of treatment trials were recorded and are thus included. One treatment trial for participant five did not record time series data; however, the remainder were successfully retrieved and so this participant’s results were retained. Collisions, time, and other derived performance values that are reported contain the manually recorded results from this trial; however the corresponding seeking metric could not be computed. For the remainder of this paper, the participant numbers will be shifted down and referred to from one to six, rather than from two to seven.

### 2.3. Experimental Protocol

Testing was performed inside a large hallway of approximately 3 m × 20 m. This environment was selected because of its availability and size, which permitted the placement of the obstacles selected for this investigation while maintaining sufficient clearance for navigation within a controlled space. The study followed an adapted alternating treatment design (ATD). The decision to use an adapted ATD approach for this study was made primarily because the methodology is well-suited to answer the questions of interest with only a small number of participants. Limiting the number of participants also offered distinct logistical advantages for this preliminary study, both in terms of recruitment and the amount of time required to conduct the study.

Subjects were asked to complete 12 m-long obstacle courses under three different conditions (A, B, and C) in three phases. Each obstacle course consisted of 5 randomly placed 63-inch inflatable vinyl columns within a hallway. The three phases were Introduction, Treatment Phase 1, and Treatment Phase 2. Each phase was composed of 8 trials. Each individual trial was limited to 3 min; if the time limit was reached, the trial was terminated and the subject was directed to reset for subsequent trials. The baseline phase was composed solely of Condition A, the introduction. In Condition A, subjects attempted to complete the obstacle course with the full use of their sight while holding an iPhone in front of them and wearing stereo headphones in transparency mode but without active assistance from EchoSee. The app still actively collected position and orientation data, but it provided neither visual nor audio feedback. Subjects were asked to complete 8 randomized configurations of the obstacle course during this phase to accustom them to wearing the headphones while holding and moving with the phone in an approximation of the experimental orientation. The second and third phases, Treatment Phases 1 and 2, consisted of one trial each from Conditions B and C in 4 random pairings, composing a total of 8 trials per phase. Condition B asked subjects to hold an iPhone and wear headphones in transparency mode while navigating an obstacle course blindfolded. This was done without the assistance of the audio soundscapes generated by EchoSee. Condition C asked subjects to perform the same task as Condition B, except with the aid of EchoSee’s soundscape. In brief, subjects completed one set of 8 sighted trials (Condition A) followed by two sets of four pairings of Conditions B and C. This resulted in a total of 24 trials for each participant: 8 sighted trials, 8 unaided blindfolded trials, and 8 aided blindfolded trials. The data reported in Section 3 comprise the latter two trial sets.

The obstacle courses were randomized under some constraints to ensure consistency between runs while also minimizing any learning effects. All 24 course configurations were generated in advance and each participant completed that same set of 24 configurations in a randomly selected order. Participants initiated each trial from the same location, regardless of Condition. To maintain timing consistency across both participants and trials, participants were instructed that the time-keeping for the trial would begin as soon as they crossed the experimental origin and that they should begin navigating the course whenever they were comfortable following the verbal signal to start. To prevent participants gaining advance knowledge of the course configuration, participants were removed from the test environment during the setup and reconfiguration of the obstacles and were blindfolded before being led to the starting position.

### 2.4. Experimental Metrics

The 3D location (x, y, z) and 3D rotation (w, x, y, z quaternion components) of the participants were recorded by EchoSee onto the iPhone’s local storage as JSON formatted entries on a per-frame basis, averaging 60 data points per second. Additionally, for each trial, the trial-relative position and orientation of the fiducial marker, the positions of the randomly placed obstacles relative to this marker, and the number of times participants made contact with these obstacles, were recorded separately. These data provided sufficient information to fully reconstruct the 3D trajectories of the participants during each trial. Each run was written to its own unique file identified by participant number and timestamp of initiation. Data were subsequently registered and processed by custom scripts developed using MATLAB [58].

These scripts used the recorded timestamps and positions to determine several derivative values aimed at quantifying the relative confidence and accuracy with which participants navigated the obstacle courses. These quantities were (1) the amount of time the participants took to complete each trial, (2) the estimated direction of travel, (3) the “seeking” exhibited by the participant, and (4) a score similar to ADREV (assessment of disability related to vision) [59] for each trial. The direction of travel is estimated from the position data by calculating the angle relative to the positive x-axis of a vector originating at one data point and ending at the next data point.

Seeking, defined as rotation in the traversal plane away from the direction of travel, was calculated using the difference between the rotation of the device about the vertical axis (the z- or yaw-axis) and the calculated travel direction. The local peak deviations for each trial were summed, yielding a single “total head turn angle” value per trial. The peaks were found by first subtracting the first-order trendline from the head turn angle data to remove drift. Next, the data were separated into two series representing left and right turns, respectively, (greater or less than zero after de-trending). The local maxima were then located and filtered such that only points with prominence greater than 7.5% of the global maximum deviation value were kept. This filtering was done to reduce jitter and to reject small-scale, gait-related oscillations. The sum of the absolute value of these peaks was then used as the “total head turn angle” or “seeking” score for that trial, reported in radians. This metric was used as a representative of the amount of environmental investigation (i.e., sweeping) performed by participants.

The total time to complete the trial was calculated as the difference between the timestamp of the first data point after the participant crossed the world origin and the first data point after the participant crossed the completion threshold (12 m forward from the world origin). The total number of collisions with obstacles in each trial was manually recorded by study personnel.

ADREV can be calculated as a quotient of the number of collisions and elapsed time, which is then traditionally scaled to lie between 1 and 8. The decision was made to use a related, but altered, distillation of collisions and time to better capture the desired outcomes (i.e., fewer collisions and less elapsed time should both change the performance score in the same direction as these are both desired metrics). To that end, a new metric is introduced called the Safety Performance Index (SPI), which is the inverse product of the elapsed time and the number of collisions plus one (to ensure there is no multiplication or division by zero).

$$\mathrm{SPI}=\frac{1}{\Delta T*\left(\mathrm{Collisions}+1\right)}$$

In this equation, ΔT corresponds to the total elapsed time of a given trial, and Collisions corresponds to the number of times the user collided with any obstacle during that trial. A greater SPI is indicative of better navigation performance (i.e., less elapsed time, fewer collisions) where a lesser SPI indicates a weaker performance. The reported SPI scores are normalized to lie between 0 and 1 for easier visualization (this normalization is done by dividing all scores by the maximum score in a given aggregation approach).

## 3. Results

### 3.1. Application Development and Testing

**Figure 6:**
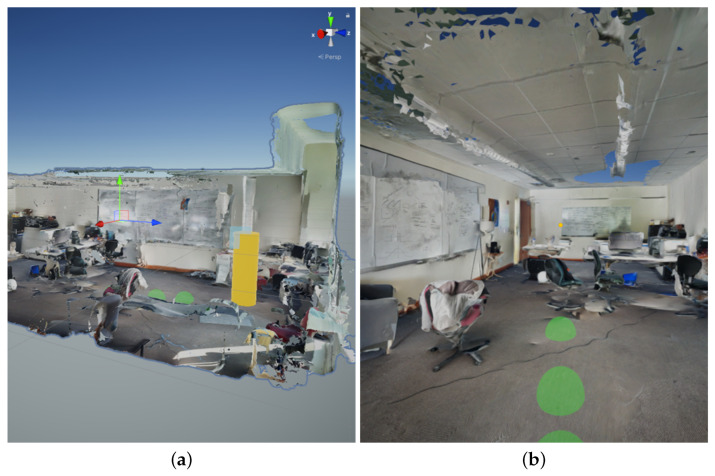
Example use of EchoSee within a digitized, real-world environment. Created using photogrammetry, the scene model was imported into the Unity game engine, permitting the EchoSee application to interact with the virtualized geometry and create audio soundscapes using raycasting. (**a**) Viewed from an external perspective, a user is represented by a yellow cylinder, also visible is the coordinate system of the virtual environment and two raycast audio sources represented as green spheres. (**b**) The virtual environment viewed from the user’s perspective, visible in the center of the image (on the back wall of the room) is a yellow point representing the focus point of the capture device, while, visible to the bottom of the image are three green spheres which represent the spatialized audio sources arranged in a preliminary configuration.

Testing of the EchoSee application during development took place within ideal virtual environments, digitized environments, and real-world environments. Initial conceptual design and development tests were done on a desktop computer within virtual environments, such as ideal corridors or simple mazes. Once a coarse design had been developed, real-world environments were digitized with technologies such as photogrammetry for more representative virtual testing. Figure 6 shows an example of the application being used to produce a soundscape of a digitized environment in the Unity engine using an early sound source configuration.

Visible to the right of Figure 6a, the user is represented by a yellow cylinder and the sound sources are represented by three green spheres (the third is located to the left of the first two and is mostly blocked by the red chair). In this preliminary configuration, only three sound sources were used in order to simplify the development process and validate the raycasting operations. The cylinder representation is not germane to the operation of the application and was used only for purposes of visualization. Figure 6b shows the virtual scene from the perspective of the capture device. The center of focus for the virtual camera in the scene is represented by a small yellow dot on the back wall of the environment. The three spheres shown in the frame (one only partially visible at the extreme bottom edge) are raycast onto the floor at their own angular offsets and play their own sounds to provide spatial audio feedback. The use of virtualized environments allowed EchoSee to be tuned without the added processing time of compiling to a mobile platform every time something was changed, greatly increasing development efficiency.

**Figure 7:**
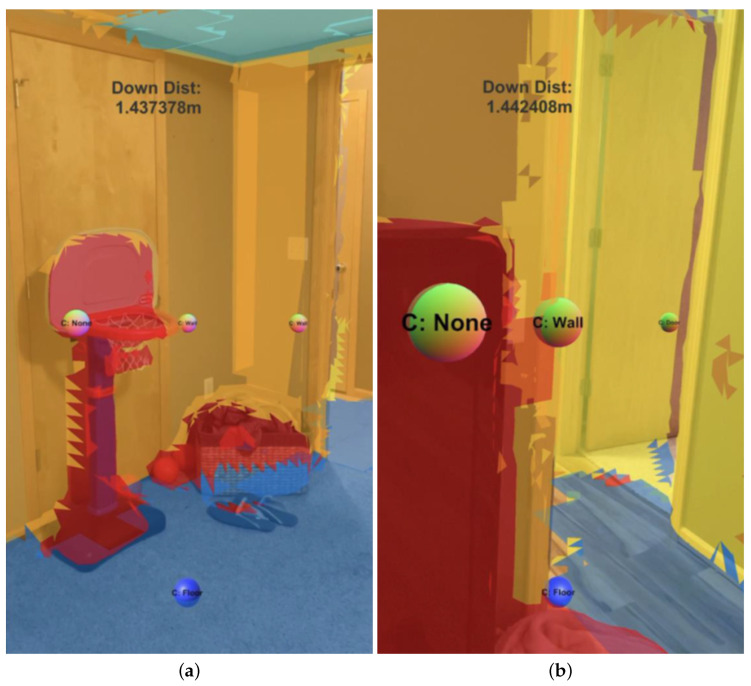
Example real-time use of EchoSee within a real-world environment produced by an early development prototype. The reconstructed 3D scene is represented with color overlays, coded according to their classification. The spatial audio sources are visualized with spheres and text labels corresponding to the surface with which they are interacting. (**a**) Medium range view of a reconstruction from a residential environment, four spheres are visible, three across the center of the image, themselves textured with a normal map, and a single sphere on the floor, colored blue. The current distance to the room’s floor from the device is labeled as ‘Down Dist’ and is approximately 1.44 m. (**b**) Close range view of a scene, where the virtual spheres’ size across the middle of the image change with their proximity to the device (farther being smaller and vice versa).

Once the design and implementation had undergone initial testing in purely virtual environments, the development was continued using a mobile device within real-world environments in preparation for the execution of the described study protocol. Figure 7 shows an example real-world use of EchoSee within a living room setting. An updated configuration of sources is shown, comprised of four sources, three across the center of the scene (one offset to each side and one in the middle) with the fourth at an angular offset down from the center. The color texturing that is visible in the scene shows the classification of the different surfaces produced by ARKit. The surface classification is also represented by the text labels on the audio sources (i.e., “floor” on the bottom source in blue, and “none”, “wall”, and “door” from left to right, respectively, in Figure 7b).

At this point in the development, a decision had to be made regarding the placement of the audio sources within the scene. Several prior works, including [39,40,43,60] (as described in Section 1), had sought to use various types of virtual reality techniques to sonify virtual environments, or in the case of [46], to use custom hardware to sonify real environments. All of these works had to select a methodology for sonifying their respective environments. Because two of the fully virtual methods [39,43] were in complete control of the environments for their implementations, the virtual objects were themselves designed to play audio signals (variously selected as musical “humming” [43], object-type specific sounds, or “metal pinging” [39]). For the other virtual implementation [40,60], a large grid of sources (7×13 objects with a field of view spanning $30^{\circ }\times 50^{\circ }$ vertically and horizontally, respectively) was projected into the scene using a kind of raycasting where each source played “impact sounds.” These techniques produced positive results, as reported in those works, but have only been used with comprehensive prior knowledge of the operating environments. In ref. [46], the “Naviton” sonification approach was performed on a per-detected-object basis determined by the objects’ intersection with a virtual plane sweeping forward from the custom scanning device at predetermined time intervals. When the intersection of an object with this sweeping plane was detected, a sound of variable pitch and amplitude (both related to the object’s distance) and duration (related to the object’s width) was played at the intersection location(s). That same work’s “Sound of Vision” project implements additional sonification techniques, one of which operates similarly to “Naviton” but instead sweeps from side to side; another subdivides the field of view into three regions (left, right and center, all $30^{\circ }$ wide) and plays spatialized signals for the closest object detected in each region.

When considering how to sonify real environments, all these approaches from prior works were considered and it was determined that a synthesis of these techniques would best serve the objectives and coordinate with the employed technologies of EchoSee. Because complete environmental knowledge is not available to EchoSee in real-world environments (as the application is dynamically building a map while the user traverses through their environment) the a priori placement of sound sources used by the majority of the fully virtual methods described was not possible. Further, as the mesh built by EchoSee is a single semi-continuous object, the per-object sounds produced by “Naviton” and “Sound of Vision” were not appropriate. Rather, a mixture of these techniques was developed, one that used the musical “humming” of [43], the raycast sources of [40,60], and the regionally segmented sounds of [46].

The decision to use a limited number of discrete sources was made to maintain both consistent software performance and soundscape intelligibility. The configuration of sources (which Section 2.1 describes in detail) was chosen to give reasonable coverage of the environment in what were considered to be important regions. The center source (offset $0^{\circ }$, note G4 [392 Hz], as shown in Figure 4), communicates “range-to-target” information. The left and right sources (offsets of $30^{\circ }$, note G4 in Figure 4) were to provide “finger trailing” input, similar to holding one’s hand out to touch a wall, or alternatively as a substitution for peripheral vision. The upper source (offset $20^{\circ }$, note B4 [493.88 Hz] in Figure 4) is to give feedback regarding potential overhanging obstacles. The lower two sources (offset $30^{\circ }$ and $60^{\circ }$, notes E4 [329.63 Hz] and C4 [261.63 Hz], respectively, in Figure 4) are to inform users about tripping hazards and imminent elevation changes.

### 3.2. Developmental Testing of Analytics

Once the operation of EchoSee and the configuration of sound sources was established, the analysis framework for the performance metrics had to be tested. As described in Section 2.1, the values directly recorded by EchoSee at 60 FPS were its position, rotation, the timestamps of those samples, and the location and orientation of a fiducial marker. The performance of EchoSee is demonstrated below in three environments, an empty hallway, a hallway with an obstacle, and a stairwell. Once the demonstrations had been performed and recorded, the logged information was imported into MATLAB and parsed into native data structures. The first processing step after ingesting the data was alignment. This alignment was accomplished by first extracting the position of the fiducial marker within each session’s arbitrary coordinate system. That position defined the translation necessary to shift the recorded data to a common origin. Once translated, the recorded position and orientation data were still misaligned. A series of transforms had to be determined to realign the disparate coordinate systems of the different datasets. These transformations were calculated using the relative orientation of the fiducial marker and the unit X and Y vectors of the desired absolute system (X and Y together a constrain the Z direction). The difference in angle between the absolute and relative X axes was taken and a transform calculated to rotate the relative to the absolute. This transform was then applied, but a single-axis alignment is insufficient to fully define the rotational transform of a coordinate system. To complete the alignment, this process was repeated for the relative Y axis. These transforms were sequentially applied to all the position and orientation data. This translation and alignment process was performed independently for each session’s recorded data.

**Figure 8:**
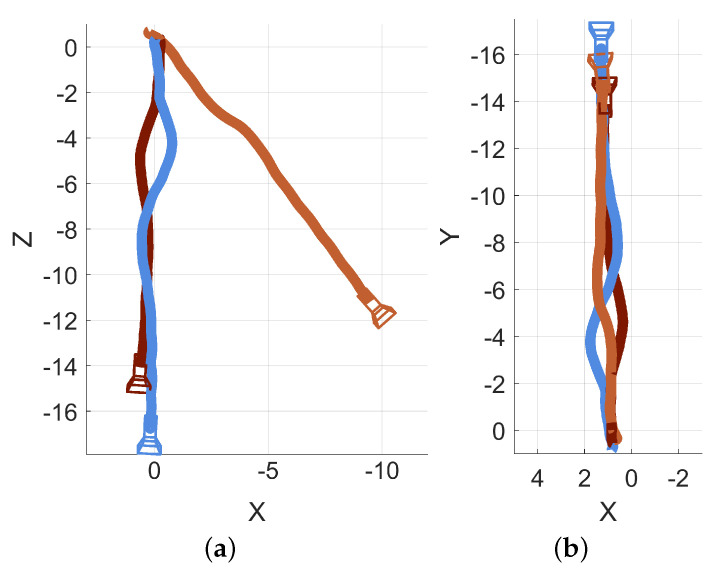
Demonstration of the alignment inputs and outputs. All units are in meters. (**a**) Unaligned data which, although recorded in the same hallway with similar start and end points, do not share a common coordinate system because each 3D reconstruction was generated during a different session with its own initial, arbitrary coordinate system. (**b**) Aligned data that shares the same fiducial marker, allowing the traces to be translated and rotated into a common coordinate frame.

Three sessions were recorded in an open hallway to demonstrate the necessity and effects of this alignment procedure. All three sessions were started and ended at similar real-world locations, with the fiducial marker remaining in the same position for all three sessions. An overlay of all three path traces from the unmodified sessions is presented in Figure 8a. An overlay of the same three sessions after their respective alignments are applied is presented as Figure 8b. Some of the unaligned datasets show substantial differences while others are relatively consistent. There is sometimes no need to perform transformations for data series to be aligned with each other (evident in the blue and dark red series in Figure 8a). However, as part of the alignment process, the coordinate frame is reoriented to have a more conventional configuration (as demonstrated by the change from Z,X axes in Figure 8a to the X,Y axes in Figure 8b).

**Figure 9:**
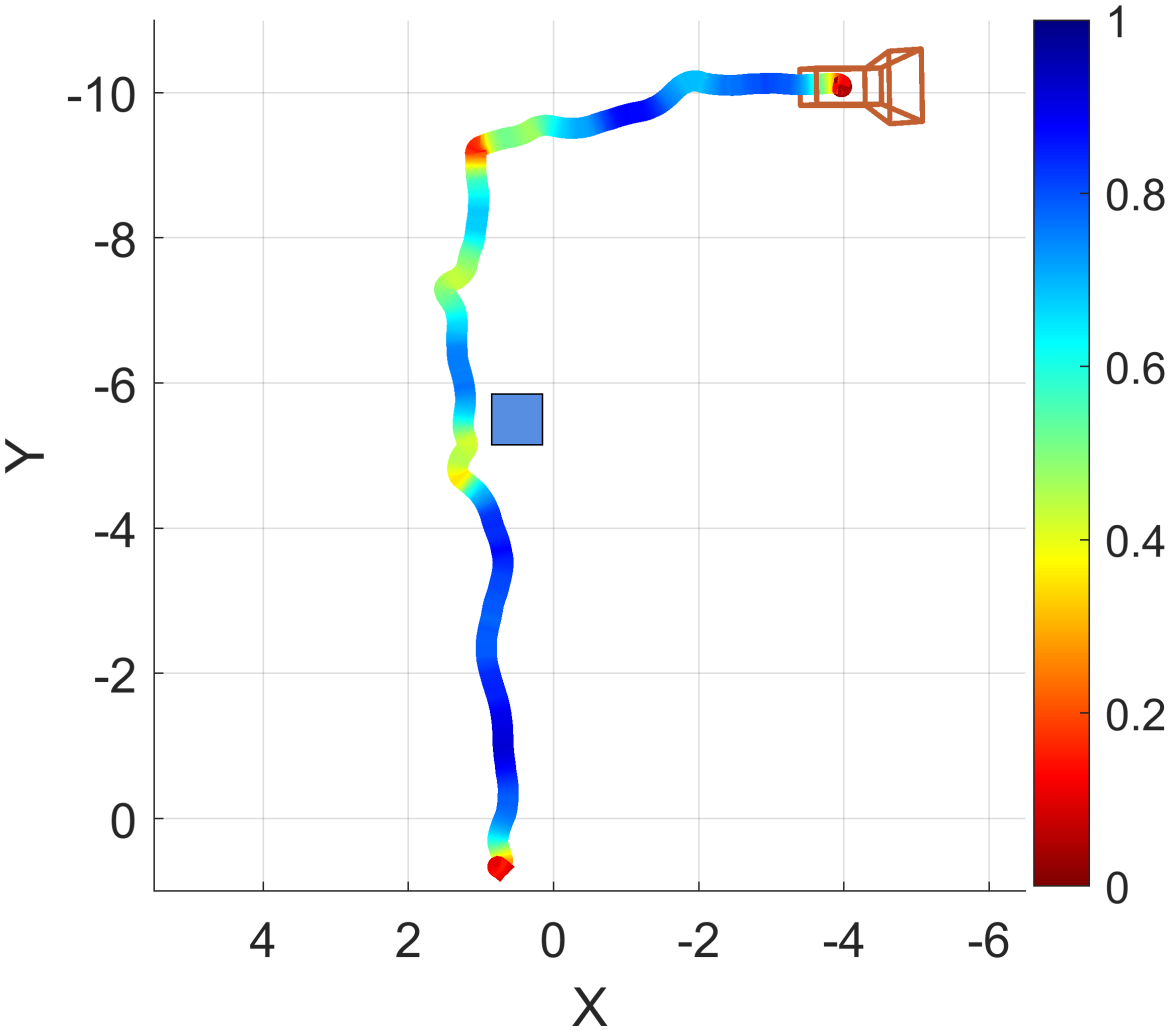
An example operation of EchoSee viewed as if looking down on the hallway from above. The blue box represents the real-world position of an obstacle. The color coding of the trace indicates the normalized traversal speed at that point, red being slower, blue being faster. Units are in meters.

Another example navigation session was conducted in which the operator walked forward through a hallway, navigated around an obstacle, turned right, and moved down another hallway. A path trace visualization of this example is provided as Figure 9. The path trace is shown with a box plotted in the approximate real-world position of the obstacle (a recycling bin). Encoded in the trace color is the normalized traversal speed, red indicating slower traversal and blue indicating faster traversal.

**Figure 10:**
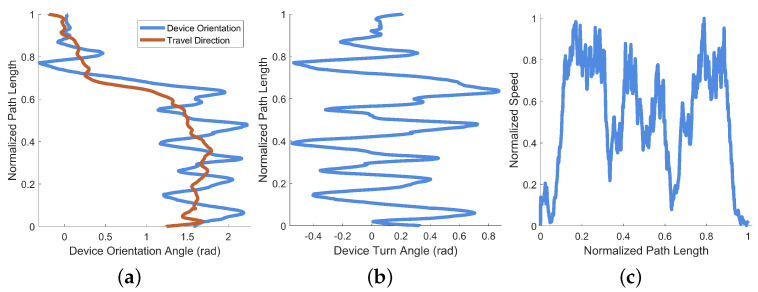
Performance metrics derived from an example operation of EchoSee. (**a**) Device turn angle and the approximate direction of travel against normalized path length, note the correspondence between (**a**) and the path trace in Figure 9, where the sharp decrease in device angle of π/2 radians matches the right turn in the path trace. (**b**) Device deviation angle calculated as the difference between the two curves in (**a**) plotted against normalized path length. These data were used to calculate the total head rotation angle as approximately 1.99π radians. (**c**) Normalized speed plotted against normalized path length.

A plot of the device orientation angle and estimated travel direction from this example is provided as Figure 10a (their difference is also shown as deviation angle in Figure 10b). A plot of the normalized travel speed is provided as Figure 10c. Device orientation is plotted against the normalized path length of the example session in Figure 10a, along with the direction of travel (which is estimated from the position data). Though all three axes of rotation are recorded by EchoSee, the relevant rotation data for this example were observed to be mostly contained within the rotation about the Z axis, thus its selection for plotting. The plot of the device deviation angle is presented as Figure 10b. Device deviation was calculated as the difference between head (i.e., device) orientation and approximate travel direction. Oscillations or “seeking” off-axis from the direction of travel are evident in the plot, cumulatively composing the final total head rotation value of approximately 1.99π radians (or about $359^{\circ }$). This was calculated as the sum of the absolute value of each prominent extremum (the absolute sum of the maxima and minima of the smoothed head angle deviation data).

**Figure 11:**
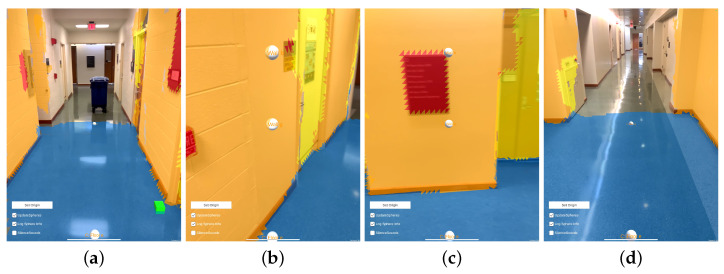
Representative frames from a screen recording of EchoSee being operated in a hallway. In all frames, the $30^{\circ }$ downward offset sphere playing tone E4 is visible directly at the bottom and the $0^{\circ }$ offset is visible at various ranges directly in the center, with the $20^{\circ }$ offset also visible in frames (**b**,**c**). Frames from (**a**) the beginning, (**b**) one third through, (**c**) two thirds through, and (**d**) the end of the session.

**Figure 12:**
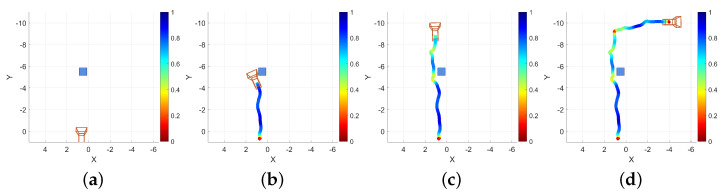
Matched path trace renderings for each of the four frames presented in Figure 11 as viewed from above. Traces with the camera at locations from (**a**) the beginning, (**b**) one third through, (**c**) two thirds through, and (**d**) the end of the session. The coloring of the trace represent normalized speed, blue is faster, red is slower. Units are in meters.

A screen capture was taken of the device while this example data was being collected. Four representative frames from that capture are presented as Figure 11. The first frame (Figure 11a) is taken from the beginning of the recording, the different classifications that are applied to the floor and walls are apparent by their blue and orange texturing, respectively. Two audio sources are visible, represented by raycast spheres, the $0^{\circ }$ offset sphere in the center of the image at a distance down the hallway and the $30^{\circ }$ down offset sphere visible at the bottom of the frame with a larger apparent size due to its proximity. The second frame (Figure 11b) is taken from approximately one third of the way through the recording, just as the operator navigates to the left around the obstacle. The yellow coloring to the right of center is a correctly classified door. Furthermore, visible in Figure 11b are three spheres at different positions relative to the capture device than those shown in Figure 11a. These differences are again due to the dynamically updated mesh environment, which causes the sources’ governing raycasts to update. The third frame (Figure 11c) is taken from approximately two thirds of the way through the recording, as the operator navigates to the end of the hallway and is about to turn right. The same three sources are visible in this frame as in the previous, but again, in slightly different positions, reflecting their response to the altered scene geometry and the user’s position within the scene. The final frame presented for this example (Figure 11d) is taken from the end of the recording, after the operator has turned to the right at the end of the first hallway and walked a short distance. Path trace visualizations were created for each of the frames presented in Figure 11; the position of the camera model correspond to the position of the capture device in each extracted frame. These path traces are shown in Figure 12 and labeled equivalently to the frame in Figure 11 that they represent.

**Figure 13:**
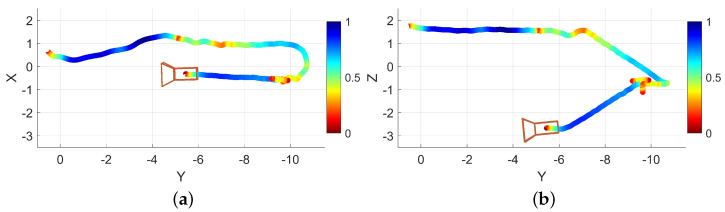
Trace of the example stairwell operation viewed (**a**) as if looking down on the stairwell from above and (**b**) as if standing to one side of the stairwell. The color coding of the trace indicates the normalized traversal speed at that point, red being slower, blue being faster. Units are in meters.

Another example is presented and analyzed where a recording is made using EchoSee while navigating into and down a stairwell. Two views of the path trace are presented in Figure 13, one from overhead, and one from the side to highlight the elevation change.

**Figure 14:**
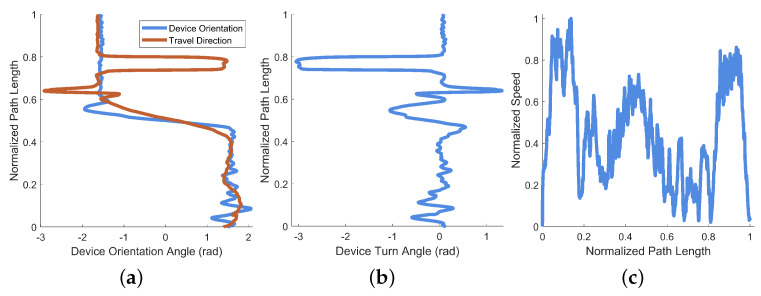
Performance metrics derived from an example of navigating a stairwell. (**a**) Device turn angle and the approximate direction of travel against normalized path length. Note the sharp decrease in device angle of π radians matches the right turn down and around the stairwell present in Figure 13a. (**b**) Device deviation angle calculated as the difference between the two curves in (**a**) plotted against normalized path length. These data were used to calculate the total head rotation angle as approximately 2.56π radians. (**c**) Normalized speed against normalized path length.

For this example data, recorded in a stairwell, the amount of “seeking” was calculated as 2.56π radians ($461^{\circ }$). Visible in both Figure 14a,b at approximately 80% along the normalized path length is a large angular shift with a plateau followed by a shift back of equivalent magnitude. This shift-and-hold is caused by a reversal of movement direction by the recording device. This reversal is demonstrated by the computed device turn angle in Figure 14b, where the plateau is π radians shifted from the previous orientation (equivalent to an “about face” as the user turns on the stairs).

**Figure 15:**
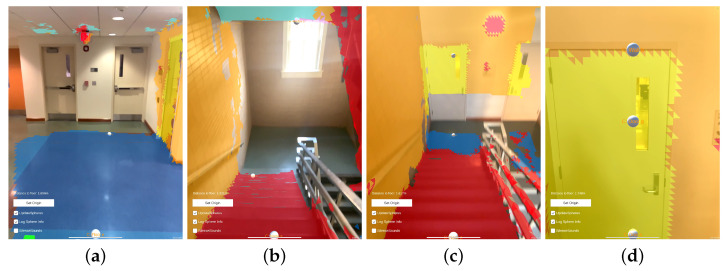
Representative frames from a screen recording of EchoSee being operated while navigating down a stairwell. In all frames, the $30^{\circ }$ downward offset source playing tone E4 is visible directly at the bottom and the $0^{\circ }$ offset source is visible at various ranges in the center, with the $20^{\circ }$ offset source also visible in frames (**b**–**d**). Frames from (**a**) the beginning, (**b**) one third through, (**c**) two thirds through, and (**d**) the end of the session.

As with the hallway example, the recorded data from this example session included the internal data from EchoSeee and a screen capture. Again, four representative views are shown from that screen capture, the first from the beginning of the session, and the second two from approximately one third and two thirds of the way through the session, respectively, with the final frame being taken from the end of the session. These frames are presented as Figure 15. The first frame (Figure 15a) shows the approach to the doors at the top of the stairwell, beyond which is a small landing. The second frame (Figure 15b) is a view from the top of the first set of stairs, just through the doors. Note the correct classification of the stairs in this frame as “other”, rather than as “floor”, which is denoted by their red mesh. Furthermore, note the deviation of the $0^{\circ }$ offset sphere from the center of the frame as it moves in response to the changing geometry which is encountered by its controlling raycast. The third frame (Figure 15c) shows a view down the second set of stairs after making a turn to the right, around the landing visible at the center of Figure 15b. The fourth frame (Figure 15d) is a view of the doorway out of the stairwell and onto the lower floor. Note the correct classification of the door in yellow and the three spheres down the middle of the frame showing the positions of the upper, central, and lower audio sources, respectively. While there are six spheres emitting sound, three of them are not visible due to the limited field of view of the device, namely, the left and right $30^{\circ }$ offset spheres and the $60^{\circ }$ downward offset sphere.

### 3.3. Analytic Results of Study Protocol

After EchoSee’s initial development, finalization of the study protocol, and recruitment of study participants, the study was conducted following the protocol described above in Section 2.3. In every sighted trial, the participants predictably moved with confidence and alacrity, adeptly avoiding all obstacles. Across the 40 sighted trials there were 0 collisions. During the treatment runs, participants demonstrated the ability to navigate the obstacle course with varying levels of performance. The relative performance in the sighted condition deviated starkly from that of either of the two treatment conditions. As the intent of this study is to determine the benefit provided by EchoSee, if any, to persons with visual impairment, the differences between the treatment conditions were deemed the most relevant to the questions at hand. Therefore, the remainder of the analysis is devoted to the relative performance between treatment conditions.

**Figure 16:**
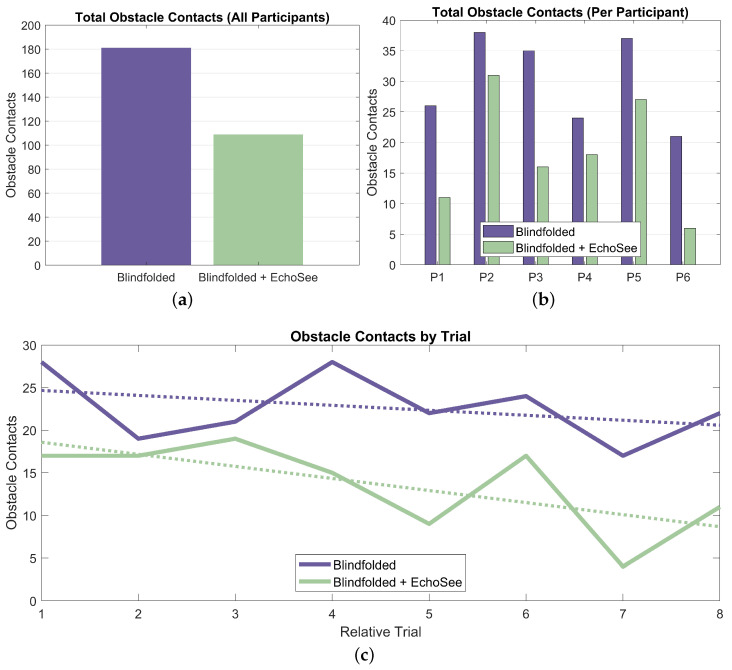
Graphs of the obstacle contacts recorded during the feasibility study. (**a**) The total contacts recorded for both of the two trial conditions. (**b**) The total contacts separated by participant and trial condition. (**c**) The total contacts recorded during each trial run, listed in the order in which they were encountered, separated by trial condition. The slope for the unassisted trendline is −0.58; however, for the assisted trendline it is −1.42.

The number of collisions was collated over the 16 Treatment Phase 1 and 2 trials for the six participants. For Condition B (blindfolded unassisted), there were 181 total contacts and under Condition C (blindfolded with assistance), there were 109 total contacts as presented in Figure 16. These results show a substantial reduction in the number of contacts recorded with the assistance of EchoSee compared to without it. This advantage holds both in aggregate, and on a per-participant basis as shown in Figure 16a,b. There is also evidence of learning effects, demonstrating that the participants were improving their performance over time. These effects are present for both the unassisted and assisted trial conditions, however the rate of improvement (the slope of the trendline) is steeper for the assisted case (−1.42 collisions per trial) as compared to the unassisted case (only −0.58 collisions per trial).

**Figure 17:**
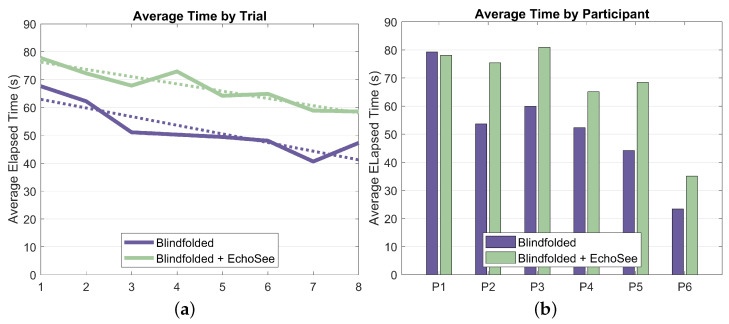
Elapsed time, aggregated across trials and participants, respectively. (**a**) Elapsed time aggregated across trials separated for both of the two trial conditions. The unassisted trendline has a slope of −3.10, and the assisted trendline has a slope of −2.60. (**b**) Elapsed time separated by participant and trial condition.

Elapsed time was another key recorded metric for the study trials. The aggregated elapsed time results are presented as Figure 17. On average, participants navigated more slowly when using EchoSee. The average completion time for the unassisted trials was 50 s, while the average assisted completion time was 67 s. Completion times trended downward for both trial conditions, again demonstrating that the participants were becoming more comfortable operating blindfolded. The slope of the unassisted trendline is −3.10 s per trial and the slope of the assisted trendline is −2.60 s per trial. The shallower slope for the assisted trials as compared to the unassisted trials could indicate that while participants were learning and acclimatizing to blind navigation in both conditions, they were taking time to interpret the soundscapes produced by EchoSee rather than feeling out their environment with their hands or risking an unexpected encounter with an obstacle.

**Figure 18:**
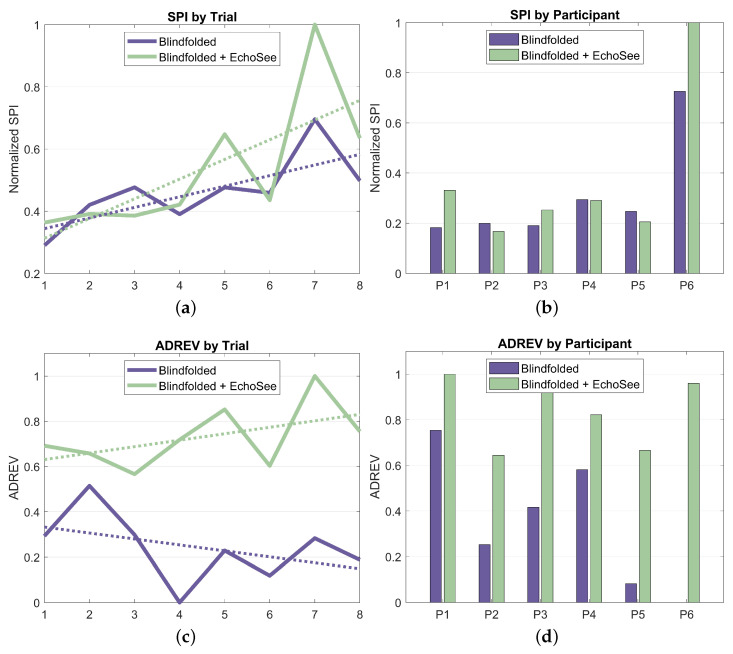
Normalized SPI and ADREV derived from obstacle contacts and elapsed time, aggregated across trials and participants, respectively. (**a**) SPI aggregated across trials plotted against trial number for both of the two trial conditions. The unassisted trendline has a slope of 0.017 and the assisted trendline has a slope of 0.067. (**b**) SPI separated by participant and trial condition. (**c**) ADREV aggregated across trials plotted against trial number for both of the two trial conditions. The unassisted trendline has a slope of −0.0262 and the assisted trendline has a slope of 0.0285. (**d**) ADREV separated by participant and trial condition.

With its constituent metrics in place, SPI and ADREV may now be examined; they are shown in Figure 18. The trends in SPI show a nominal improvement in the unassisted case over the course of the study and a noisier, but more substantial, upward trend in the assisted cases. The variability in the assisted curve in Figure 18a is due, in large part, to the inter-trial variation in the number of obstacle collisions, which also had larger inter-participant variation. Elapsed time was also observed to have increased intra-participant and inter-trial variation in the assisted cases, further increasing the variability compared to the unassisted curve. When using EchoSee, participants were likely to pause and investigate if faced with strong, unexpected feedback (such as when a participant was “stuck” between a wall and an obstacle). This behavior seems to be attributable to the additional, and still relatively unfamiliar, source of information in the soundscapes provided by EchoSee. Such investigation generally increased the amount of and variability in time that was taken to navigate in the assisted trials, but also contributed to a concomitant decrease in the number of collisions that were sustained.

ADREV shows a similar trend in the assisted case, with a small, but appreciable upward trend in performance. In the unassisted case, however, the performance has a slight downward trend. This may be somewhat confusing given that both ADREV and SPI are composed of the same metrics (elapsed time and collisions). This difference in output between the two performance metrics draws to the fore the main perceived drawback of ADREV. Since it is, at its core, a simple ratio of collisions and time, there are sometimes ambiguous or misleading outcomes. Take the extreme hypothetical examples of a user sprinting through the course and hitting all of the obstacles as compared to a user arbitrarily pausing for several minutes in the middle before completing and having only a couple collisions. The first example would have a relatively higher ADREV score because of their faster completion time combined with more collision. In the second example, the user’s extreme traversal time would overshadow their relatively low number of obstacle collisions, causing a much lower ADREV score. Although in this study, the users were not colliding with obstacles deliberately and were also working to finish the trials as quickly as was comfortable, the metric is not without merit. However, the ambiguity of the metric renders explanations of trends such as those present in the per-trial ADREV plot, Figure 18c, unsatisfactory. This interpretive difficulty accentuates the conflicting impetuses of collisions and traversal time, which led to the introduction of SPI, a more easily explainable quantification of the constituent factors, as described above.

**Figure 19:**
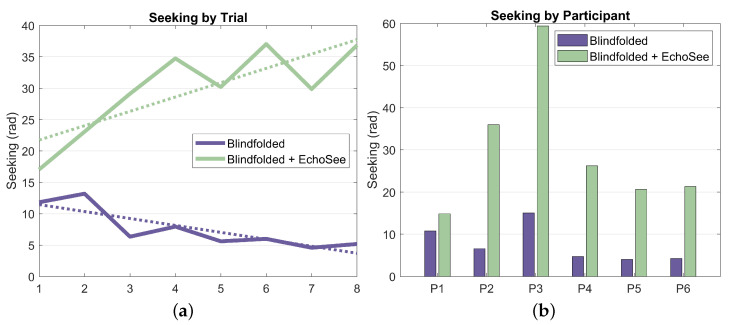
Seeking value calculated from the head turn angle data, aggregated across participants and across trials, respectively. (**a**) Seeking aggregated across trials plotted against trial number for both of the two trial conditions. The unassisted trendline has a slope of −1.11 and the assisted trendline has a slope of 2.28. (**b**) Seeking value separated by participant and trial condition.

The final metric that was analyzed was the amount of seeking, or the cumulative head turn angle. This metric, averaged per participant and per trial, is presented below as Figure 19 (note that position and rotation time series results from the fifth unassisted trial for participant 4 were not recorded; thus, analysis of the seeking behavior was not possible and, therefore, not included). These results show a stark difference between the behaviors exhibited by the participants in the two blindfolded trial conditions. Across all participants and trials, there is a marginal to multiplicative increase in seeking when EchoSee is in use. This behavior does exist in the unassisted cases, but the observed learning effect is towards a steady decrease in seeking, as compared to the quick ramp and then slower increase visible in the assisted cases. These differences may be explainable by the derived benefit from engaging in seeking under the two different conditions. When unassisted, the participants gained little from sweeping the device back and forth, or seeking, and evidently did so only sparingly. In contrast, when assisted by EchoSee, the participants seem to learn that the application provides substantial benefit, and so seek liberally. The initial difference in the two curves is most likely attributable to the initial description of EchoSee’s intended use, which included a brief example of a perfunctory sweeping motion with the device. As the data indicates, the participants seem to have mimicked the demonstrated behavior and then quickly increased until reaching a plateau, which could be caused by information saturation. It is possible that, with additional testing, conclusions about the optimal amount of seeking could be made.

### 3.4. Qualitative Results of Study Protocol

Following the conclusion of their involvement, participants were asked to fill out a survey relating to their experiences during the study. The main objective of the survey was to ascertain any differences in feelings of safety and confidence experienced by the participants while they were or were not aided by the EchoSee application when blindfolded. Some additional questions were asked to gauge the level of satisfaction that users felt with the current implementation of the soundscape. Participants were asked to rate all seven questions on a scale from one to six (where one was Strongly Disagree and six was Strongly Agree). The average responses were calculated using a normalized scale, with the minimum being 0 and the maximum being 1.

**Figure 20:**
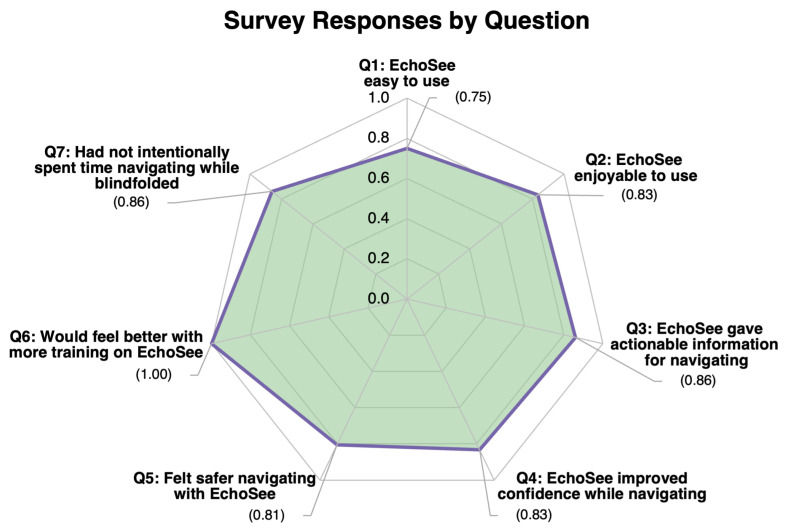
Graphical results for the post-study perception survey. Responses from the six participants were normalized between 0 and 1 and then averaged.

**Figure 21:**
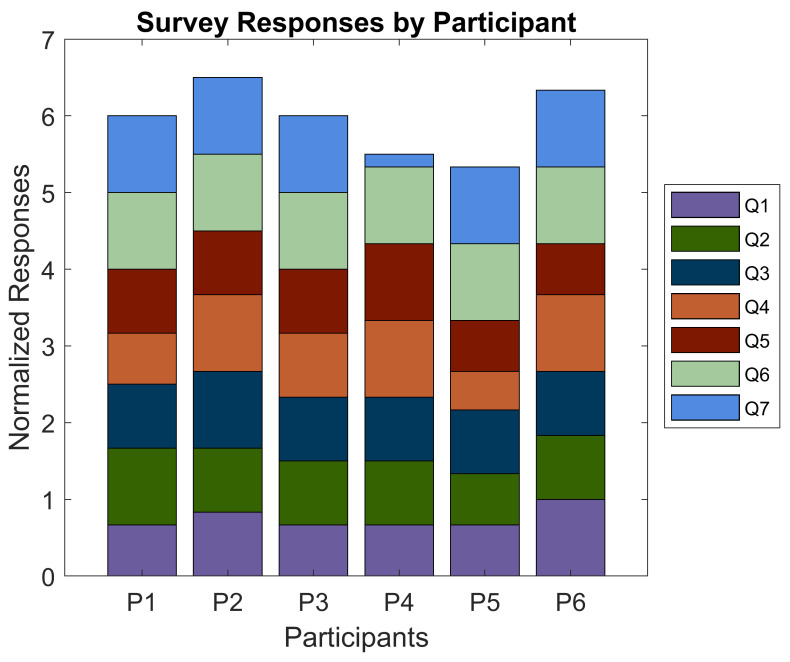
Graphical results for the post-study perception survey when rated from 1 (score of 0) to 6 (score of 1) for each participant. Q1: This application was easy to use. Q2: The application was enjoyable to use. Q3: This application provided actionable information for navigation while blindfolded. Q4: This application improved my confidence in navigating while blindfolded. Q5: I felt safer when navigating using the application while blindfolded. Q6: I feel that I would be able to perform better if I had more training with the application. Q7: Prior to this study, I had not intentionally spent time navigating while blindfolded.

The first two questions aimed to ascertain participant satisfaction: (Q1) “this application was easy to use” and (Q2) “the application was enjoyable to use”. Among the six study participants, the averaged score of the first question was 0.750 and for the second it was 0.833. The next three questions: (Q3) “this application provided actionable information for navigation while blindfolded”, (Q4) “this application improved my confidence in navigating while blindfolded”, and (Q5) “I felt safer when navigating using the application while blindfolded” were posed to gauge the degree to which participants’ performances correlated with their perceived performance. These questions received average scores of 0.861, 0.833, and 0.806, respectively. The sixth question, “I feel that I would be able to perform better if I had more training with the application”, was expected to correlate with the appearance of learning effects in the EchoSee-assisted cases. A positive correlation was indeed observed with the unanimous rating of the question at 1.00. The final question, “Prior to this study, I had not intentionally spent time navigating while blindfolded”, was posed to establish if there was an underlying explanation for any outliers. Only participant 4 reported spending any time intentionally navigating blindfolded. When queried regarding their experience the participant estimated that a cumulative total of five hours had been spent. This experience was not recent to the trial and was deemed by the researchers to not have substantive impact on the participant’s performance. Figure 20 visualizes the average response to each survey question. A graph of the color-coded responses by each participant to each question is presented in Figure 21.

### 3.5. Results Summary

The development, testing, and initial user evaluation of EchoSee yielded significant implications across multiple domains. Section 3.1 demonstrated the successful implementation of a real-time 3D audio soundscape on a mobile platform, utilizing advanced 3D scanning and AR technologies. Section 3.2 described EchoSee’s ability to record usage data and provide a variety of metrics related to a user’s navigation performance. Section 3.3 presented the quantitative results from the feasibility study, showing improvements in obstacle avoidance and indicating users become increasingly proficient after only a few navigation trials. Section 3.4 provided qualitative user feedback, indicating positive perceptions of EchoSee’s usability and potential.

Below is a summary of this work’s results in terms of its technological contributions:

- **Real-time 3D Soundscape:** Successfully implemented EchoSee, a system generating real-time 3D audio soundscapes on a mobile device by integrating real-time 3D scanning, AR technologies, and spatial audio.
- **Analytical Framework:** Developed methods for aligning and extracting various metrics from 3D trajectory data that are relevant to a user’s navigation performance.

Results as they relate to supporting enhanced navigation for PVI can be summarized as:

- **Obstacle Avoidance:** Demonstrated a 39.8% reduction in obstacle contacts when using EchoSee (181 unassisted vs. 109 assisted).
- **Environmental Awareness:** Observed substantially increased seeking behavior (slope of 2.28 assisted vs. −1.11 unassisted), indicating improved environmental investigation.
- **Navigation Behavior:** Noted a 34.0% increase in average completion time (50s unassisted vs. 67s assisted). This additional time suggests more thorough explorations with the benefit of reduced collisions.
- **SPI & ADREV Performance:** EchoSee-assisted navigation showed improved trends in both the SPI and ADREV metrics.
- **User Perception:** Recorded high user ratings for EchoSee providing actionable navigation information (0.861/1.0) and improved navigation confidence (0.833/1.0).
- **Learning Effects:** Observed increased learning rate within assisted conditions for collision reduction, suggesting navigation benefits with EchoSee after only a few trials.

## 4. Discussion

The outcomes of this study demonstrate the viability of the presented navigational assistance application, EchoSee. Participants were able to improve their blindfolded navigation performance, as described by several objective metrics. The application reliably performed as it was intended, both in its generation of the soundscapes to assist blindfolded participants navigate during the relevant trials, and in its role as a data recorder (the reported data losses were the result of user error). The data that were recorded allowed the trajectories of participants to be reconstructed and analyzed in full six-degree-of-freedom space (translation and rotation in each of the three Cartesian axes, x, y, and z). The technical performance of the platform, and the results of the feasibility study, offer impetus for the further development and expanded study of EchoSee.

### 4.1. Development of EchoSee

EchoSee is currently implemented on commercially available iOS platforms (off-the-shelf iPhones and/or iPads) that are equipped with LiDAR scanners and also leverages AirPods Pro stereo headphones to play the generated soundscapes. While there are many possible sonification techniques (such as those described in Section 1 and Section 3.1), the approach and configuration used in the current implementation (described in Section 2.1) was selected for two reasons: (1) frame rate performance and (2) intelligibility (both interpretive and explicative). Alternate sonification methods that utilize ray-tracing for audio spatialization were explored in the very early stages of development. However, these alternatives were not pursued on the grounds of hardware incompatibility, processing constraints, and a higher threshold of comprehension, although ongoing technological advancements may motivate reevaluation of this decision in the future (see Section 4.8). Many techniques and software packages are already available for the simulation of spatial audio and the dynamic interactions of sound with complex geometries, such as the ones provided by Unity that EchoSee employs. However, the accessibility of some of these technologies on mobile platforms is quite restricted. If software will even run on a given platform, it may not run well enough to provide useful outputs. Given that one of the key objectives of EchoSee is to empower PVI using easily accessible devices that may already be in their possession, implementing features or using approaches that require non-standard modifications, rely on external services and networked hardware, or have limited performance was avoided.

### 4.2. Experimental Development

An experimental paradigm was presented for studying the feasibility of the EchoSee application. When evaluating the questions under consideration (listed at the end of Section 1), it was determined that they could all be answered to a sufficient degree using a relatively small cohort experiencing the experimental conditions. This determination provided the grounds for structuring the feasibility study using an adapted ATD. That design methodology allows for a small number of subjects to undergo multiple “treatments” and for any performance differences to be adequately analyzable. It is naturally a crucial goal of future work to expand the number of participants and study the efficacy of EchoSee with the eventual target audience, PVI.

The study was enabled by the position and orientation tracking and logging capacity with which EchoSee was designed (the principle motivation for their implementation). For purposes of ethical assurance, the decision was made to log the information locally to the iPhone rather than streaming it to a networked storage solution. During the study trials, EchoSee recorded 3D spatial and rotational information at an average rate of 60 samples per second. All told, the application recorded nearly 600 MB of raw data totaling well in excess of 330,000 spatiotemporal snapshots. The various metrics used in this study (detailed in Section 2.4) were chosen to align with those present in prior work (particularly as in [43]) and because they were determined to directly relate to study questions posed during this investigation. While EchoSee was being built and tested, it became apparent that the data being recorded by the system was not oriented in the same way during each session. As described earlier (Section 2.1), this variability in session origin is because the ARKit subsystem [56] does not have any absolute reference when it is building the mesh of the scene. Without a consistent coordinate system, direct comparison across the study trials would be difficult. One approach that was considered was to create a physical mount in a fixed position and start all sessions with the device locked to that mount. It was reasoned that if the application was initiated from the same location each time, then the generation of the mesh and associated session origin would always be in the same place. This concept was not pursued as it was determined to be too disruptive for the test participants. Rather, the post hoc registration approach described in Section 2.1 was settled upon, making use of a real-world fiducial marker that was digitally associated with a virtual-world coordinate and rotation. This association was performed on a per-trial basis, allowing each trial to be shifted and aligned with the objective origin (which was held constant for all trials).

### 4.3. Learning Effects

Participants in the presented study were only given a limited amount of instruction on how to use the soundscapes produced by EchoSee. They received only perfunctory training with the device before they were asked to navigate the aforementioned obstacle courses using the application. This was done to minimize the amount of experience subjects had with navigating while blindfolded before experiencing the study conditions, thus improving the isolation of the effects produced by EchoSee on blindfolded navigation performance. Participants were also verified to have had little or no substantial, intentional experience navigating while blindfolded. These factors serve to intensify the subjective significance of the observed results. The blindfolded performance of participants noticeably and immediately improved when they were using EchoSee’s soundscapes and this disparity only increased over the course of the study. As is evident from the learning effects and relative improvement over unassisted performance observed in this study, EchoSee shows the potential to offer PVI substantial and increasing benefits with continued use.

### 4.4. Training with Virtual EchoSee

From the simplest to the most advanced assistive navigation device, training is required for PVI to learn how to use it and become proficient. As one might expect, the appropriate training of PVI on how to use assistive mobility apps plays a crucial role in determining if an assistive app is adopted and ultimately used [61]. EchoSee’s preliminary study suggests that users learned to use the audio signals to navigate and avoid obstacles somewhat quickly; however, all participants still strongly agreed they would have performed better with more training.

By design, EchoSee is not limited to purely physical environments, nor is it constrained to have any physical inputs at all (as described at the beginning of Section 3 and in Figure 6). EchoSee can be programmed to produce soundscapes for hybrid (real environments with virtual obstacles) or purely virtual environments, which means that PVI could be trained to use the technology in safe and reproducible environments. This could be done by representing potentially hazardous real-world obstacles with virtual replicas, non-hazardous physical obstacles, or a combination of the two. Such a configuration would allow PVI to practice using EchoSee to navigate challenging scenarios in a controlled environment, safely gaining valuable, representative experience from situations that would otherwise be impractical or dangerous to encounter out in the world. This safety-first approach aims to cultivate user trust with the technology and prevent harm that could occur from unfamiliarity with a new assistive technology. This capacity to simulate hybrid or purely virtual environments using devices (iPhone and AirPods Pro) that are available off the shelf with no modification provides additional motivation for the future investigation of the platform.

### 4.5. Safety Performance Index (SPI)

The proposed SPI metric offered a way to evaluate participant performance that rewarded both more efficient (i.e., faster) and safer (i.e., fewer collisions) navigation. In its current form, SPI equally weights these two factors. However, this equal weighting may not be the most practical approach for all real-world scenarios or research questions.

Future studies may wish to provide differing weights to each of the contributing values. This adjustment could help make the metric more relevant to specific use cases or research objectives. For instance, in scenarios where safety is paramount, such as navigating in busy urban environments or around hazardous areas, a higher weight could be assigned to the collision factor. Conversely, in situations where speed of navigation is critical, such as emergency evacuations, the time factor could be weighted more heavily.

Furthermore, the optimal balance between speed and safety may vary depending on the individual user’s needs, preferences, or skill level. A more flexible SPI could potentially be tailored to reflect these individual differences, providing a more nuanced evaluation of performance. It is also worth considering that the relationship between speed and safety may not be linear. Very slow navigation might reduce collisions but could introduce other risks or inefficiencies, while very fast navigation might increase collision risk exponentially. Future iterations of the SPI could explore non-linear weighting schemes to capture these complexities.

Lastly, additional factors beyond time and collisions could be incorporated into an enhanced SPI. For example, the degree of deviation from an optimal path, the smoothness of navigation, or the user’s confidence level could all be relevant performance indicators in certain contexts. While the current SPI provides a useful starting point for evaluating navigation performance, further refinement and customization of this metric could yield more precise and context-appropriate assessments in future studies.

### 4.6. Real-World Considerations and Limitations

EchoSee could offer some benefit to PVI in its current form; however, the limits of the application have not yet been investigated. Even so, additional features could be implemented that may offer meaningful advantages, such as object detection and object-type-to-sound mapping, customizable soundscape tones, alternate probe arrangements, integration of GPS tracking for long-range precision improvement, etc. Some or all of these features may be necessary to meet the needs and accommodate the preferences of real-world users.

Discussed at length in [43], several “considerations for real-life application” have been addressed by EchoSee. At present, the portability of EchoSee is not a concern, with ever more powerful smartphones reaching near ubiquity, devices capable of implementing EchoSee are anticipated to become increasingly prevalent, although the refresh rate of the application could become an issue if future alterations to the sonification mode require additional processing power (e.g., ray-traced audio echolocation simulation with material-dependent sonic reflection, absorption, and transmission). The combination of multiple technologies within the easily accessible package of a mass-market smartphone means that even should the phone itself be perceived as somewhat bulky, the number of assistive devices that could be replaced by one (see Figure 1) would still make for a favorable trade-off.

It is recognized that many PVI already have remarkable capacity to navigate using their sense of hearing (as described in Section 1). Any attempt to augment their capacity must not come at the expense of reducing the effectiveness of their current abilities. This consideration was part of the motivation for using the AirPods Pro rather than other stereo headphones, as they implement audio passthrough in an easily accessible package. Alternatively, bone-conduction headphones (again, mentioned in [43]) could be used as they do not occlude the ear canal and thus do not distort ambient environmental information.

The goal of this study was to determine the implementation feasibility of the developed EchoSee application. As such, an adapted ATD was chosen for the study framework. Although capable of achieving sufficient discrimination to make determinations about effects in small test populations, this approach does not offer the generalized analytic nuance that a larger cohort would be able to elicit. Even with these design limitations, the results are encouraging.

The decision to use inflatable vinyl columns was made on practical grounds because they were roughly person-sized, did not pose a safety concern during the trials, and because they were easily detectable by the system. While the chosen obstacles were sufficient for the aims of the current study, they are not necessarily representative of many obstacles that PVI would almost certainly encounter (i.e., low overhangs or branches, furniture, thin poles, short tripping hazards, etc.).

### 4.7. Usability and Accessibility

While EchoSee demonstrates promising potential as an assistive technology, its ultimate effectiveness hinges on its end usability. As with many technological solutions, even the most innovative features can be rendered ineffective if the application is not accessible to its intended users. This is particularly crucial for PVI, who may rely on specific accessibility features to interact with mobile applications.

To ensure that EchoSee is as user-friendly and accessible as possible, several key accessibility features must be incorporated into its design. Foremost among these is compatibility with VoiceOver [62], the built-in screen reader available on iOS devices. VoiceOver audibly describes user interface elements as the user interacts with the screen, making it an essential tool for many PVI. To fully support VoiceOver functionality, EchoSee should use native iOS user interface elements wherever possible and provide descriptive alternative text for all interface components.

Beyond VoiceOver compatibility, several other design considerations are crucial for optimal usability: (1) large, easily targetable interactive elements to accommodate users with limited precision in their interactions; (2) avoidance of complex gesture patterns that might be difficult for users to learn or execute; (3) implementation of simple haptic feedback patterns to provide tactile cues during app navigation; and (4) integration with Siri shortcuts to allow voice-activated control of key features. These accessibility features not only make the app more usable for PVI but can also significantly reduce the learning curve associated with adopting new technology. For instance, the ability to launch EchoSee and begin receiving navigational feedback through a simple voice command like, “Hey Siri, start EchoSee in navigation mode” could greatly enhance the app’s ease of use and, consequently, its adoption rate among users.

### 4.8. Future Directions

EchoSee represents an advancement in assistive technology for PVI, addressing several limitations of existing solutions (see Table 1). Unlike many previous approaches, EchoSee combines real-time 3D environment scanning, on-device processing, and spatial audio feedback in a single, widely available mobile platform. This integration eliminates the need for specialized hardware, enhancing accessibility and reducing potential social barriers. EchoSee’s ability to function without internet connectivity ensures consistent performance across various environments. The system’s novel use of AR technology for real-time 3D mapping, coupled with its customizable soundscape generation, offers a more intuitive and adaptable navigation aid compared to 2D image sonification methods. Furthermore, EchoSee’s built-in analytical framework provides quantifiable metrics for assessing user performance, facilitating a path toward personalized training, feedback, and navigation optimization. Initial user studies have shown promising results, with reduced obstacle collisions and increased environmental exploration. These features position EchoSee as a versatile tool with meaningful potential for real-world navigation assistance. This section outlines key areas for future research and development to further enhance EchoSee’s capabilities and real-world applicability.

The presented user study is only a limited investigation of the feasibility of EchoSee. It is the intention of the authors to take the information learned from this study, tune and improve the application, and conduct an expanded user study (ideally enrolling PVI) to explore several additional aspects of the EchoSee platform. One of these aspects is the optimization of the configuration and tone outputs of the audio sources (as mentioned above in Section 4.6). The soundscapes produced by EchoSee currently operate using six audio sources playing predetermined tones (as detailed in Section 2.1). Future development and expanded user studies could investigate additional questions. Would a different number of sources improve participant performance? Are there better output signals that improve the communication of spatial information to users? Another avenue for investigation is the performance of the application in different navigational situations or objectives. How does the system perform indoors vs. outdoors? Are different soundscapes better suited to path-planning or obstacle avoidance as opposed to goal-seeking? At present, EchoSee’s performance has only been evaluated in static obstacle courses; is it able to provide sufficient information to users regarding dynamic obstacles (i.e., pedestrians, vehicles, etc.)?

One way some of these questions could be addressed is through the underlying technology used to build EchoSee (specifically ARKit [56]), which already provides for limited object classification. This capacity could be leveraged in the future to provide context-aware soundscape configurations. Additionally, modern artificial intelligence (AI) technologies—such as large language models (LLMs) and large vision models (LVMs)—can be incorporated to infer aspects of the environment difficult to sense via 3D imaging or convey via a soundscape. A current feature that has as yet gone unexplored is the capacity of EchoSee to determine the distance to the ground. This information could be incorporated within the application (and its soundscape) to detect and alert users to the presence of hazardous elevation changes such as stairs or curbs.

The realistic simulation of audio signals is an active area of research. As such, new algorithms and techniques for implementing this research are increasingly available. From improved dynamic spatialization algorithms to the generation of material dependent environmental echoes, these packages have the potential to substantially improve the contextual information available for EchoSee to leverage. Incorporating these packages in EchoSee would allow even richer audio feedback with ever more effective information to be provided, possibly increasing the awareness and independence of PVI making use of the platform. The adoption of such capabilities is of great interest for future development efforts.

Lastly, a promising avenue for future development is the integration of haptic feedback into the EchoSee platform. This multi-modal approach could significantly enhance the navigational assistance provided to users, especially those with dual sensory impairments (e.g., vision and hearing). By incorporating tactile signals, EchoSee could convey spatial information through touch, complementing or alternatively replacing the audio soundscape. This haptic feedback could be delivered through wearable devices, such as vibration packs worn on the torso or integrated into clothing [63]. The intensity and pattern of vibrations could communicate the location, proximity, and nature of objects in the environment, with different vibration patterns representing various environmental features or obstacles. This multi-modal feedback system could provide redundancy in information delivery and also offer users the flexibility to choose their preferred mode of sensory substitution based on their specific needs or environmental conditions. Future studies could explore the optimal integration of audio and haptic feedback, investigating how these two modalities can work in concert to provide a more comprehensive and intuitive navigational experience for people with visual impairments.

## 5. Conclusions

This paper has presented a novel assistive mobile application, EchoSee, aimed at improving the safety, efficiency, and independence with which PVI are able to navigate. Navigation assistance is accomplished by building a real-time 3D audio “soundscape” providing users with 3D stereo audio feedback about their surroundings. The application does this by using the LiDAR scanning capabilities of a modern mobile device to build a dynamic virtual 3D map of the user’s environment. This mapping is then used to place artificial sound sources at customizable locations throughout the scene. These spatialized sound sources are then used to play audio signals to the user via stereo headphones. A methodology was also presented that enabled the comparative analysis of data collected from different experimental trials of the developed application. Several metrics were extracted and computed, providing the foundation for the enabled comparison. These include the 3D position and orientation of the user and their derived “seeking” and “Safety Performance Index” (SPI) metrics. The results of the feasibility study that was conducted provide compelling evidence supporting the further development and study of EchoSee. Overall, the contributions of this paper motivate additional user studies aimed at optimizing the introduced technology to enhance the safety, efficiency, and independence of PVI navigation.

## Figures and Tables

**Table 1:** Summary table comparing selected technologies from the wide range of assistive research, applications, services, and techniques (Note: HMD stands for Head-Mounted Device).

	Goal	Source of Assistance	Feedback Mode	Mobile	Internet	VR/AR	Short Description
**Be My Eyes [4]**	Scene Description+	Remote Human	Spoken Descriptions	Yes	Yes	No	Users interact synchronously with real person for assistance
**Guide Dog**	Navigation	Trained Animal	Haptic	Yes	No	No	Trained service animal assists with navigation and other tasks
**Haskins 1946 [8]**	Navigation	Custom Hardware	Haptic/Auditory	Yes	No	No	Various white cane replacement devices using analog electronics
**Massiceti 2018 [43]**	Navigation	VR Headset	Auditory	Yes	No	Virtual	HMD that sonifies virtual environments for goal seeking and obstacle avoidance
**Strumillo 2018 [46]**	Navigation	Custom HMD	Auditory	Yes	No	No	Custom stereo vision HMD that sonifies scene in realtime
**Deemer 2019 [45]**	Scene Magnification	AR Headset	Visual	Yes	No	Augmented	HMD that provides options for magnifying scene for low vision users
**Grayson 2020 [44]**	People Description	Tethered AR Headset	Auditory	No	Yes (local)	Augmented	HoloLens with WiFi-connected computer locates and describes people in a scene
**The vOICe [52]**	Image Sonification	Mobile/PC App	Auditory	Yes	No	No	Images are captured “associating elevation with pitch and brightness with loudness” [52]
**Seeing AI [53]**	Scene Description+	Mobile App	Auditory/Visual	Yes	Yes	Augmented	“Harnesses the power of AI to describe people, text, currency, color, and objects” [53]
**EchoSee**	Navigation	Mobile App	Auditory	Yes	No	Both	Mobile application that sonifies environment in real time using onboard 3D sensors.

## Data Availability

The original contributions presented in the study are included in the article.

## Abbreviations

The following abbreviations are used in this manuscript:

ADREV	Assessment of disability related to vision
AI	Artificial intelligence
AR	Augmented reality
ATD	Alternating treatment design
ETA	Electronic traversal aid
FPS	Frames per second
HMD	Head-mounted device
LiDAR	Light detection and ranging
PVI	People with vision impairments (people who are blind or vision impaired)
SPI	Safety performance index
SSD	Sensory substitution device
VR	Virtual reality
